# The Role of Lumbar Sympathetic Nerves in Regulation of Blood Flow to Skeletal Muscle during Anaphylactic Hypotension in Anesthetized Rats

**DOI:** 10.1371/journal.pone.0150882

**Published:** 2016-03-21

**Authors:** Jie Song, Mamoru Tanida, Toshishige Shibamoto, Tao Zhang, Mofei Wang, Yuhichi Kuda, Yasutaka Kurata

**Affiliations:** 1 Department of Physiology II, Kanazawa Medical University, Uchinada, 920–0293, Japan; 2 Department of Anesthesiology, China-Japan Friendship Hospital, Beijing, 100029, China; 3 Department of Colorectal and Hernia Surgery, The Fourth Affiliated Hospital of China Medical University, Shenyang, 110032, China; 4 Department of Diabetes Surgery, The Fourth Affiliated Hospital of China Medical University, Shenyang, 110032, China; Harbin Medical University, CHINA

## Abstract

During hypovolemic shock, skeletal muscle blood flow could be redistributed to vital organs via vasoconstriction in part evoked by activation of the innervating sympathetic nerve activity. However, it is not well known whether this mechanism operates during anaphylactic shock. We determined the femoral artery blood flow (FBF) and lumbar sympathetic nerve activity (LSNA) mainly regulating the hindquater muscle blood flow during anaphylactic hypotension in anesthetized rats. Anesthetized Sprague-Dawley rats were randomly allocated to the following groups (n = 7/group): (1) non-sensitized, (2) anaphylaxis, (3) anaphylaxis-lumbar sympathectomy (LS) and (4) anaphylaxis-sinoaortic denervation (SAD) groups. Anaphylaxis was induced by an intravenous injection of the ovalbumin antigen to the sensitized rats. The systemic arterial pressure (SAP), heart rate (HR), central venous pressure (CVP), FBF and LSNA were continuously measured. In the anaphylaxis group, LSNA and HR increased, while SAP and FBF decreased after antigen injection. In the anaphylaxis-SAD group, LSNA did not significantly change during the early phase, but the responses of SAP and FBF were similar to those in the anaphylaxis group. In the anaphylaxis-LS group, both FBF and SAP decreased similarly to the anaphylaxis group during anaphylactic hypotension. These results indicated that LSNA increased via baroreceptor reflex, but this sympathoexcitation or LS did not affect antigen-induced decreases in FBF or SAP. Lumbar sympathetic nerves are not involved in regulation of the blood flow to the hindlimb or systemic blood pressure during anaphylactic hypotension in anesthetized rats.

## Introduction

Anaphylactic shock is potentially life threatening, but the pathophysiology has not been fully clarified. During hemorrhagic shock, cardiac output is preferentially directed toward vital organs. Myocardial and cerebral perfusions are indeed maintained, whereas perfusion of liver, intestines, kidneys, and muscles is significantly reduced due to vasoconstriction [1, 2]. Actually, during hemorrhagic shock, the sympathetic nerve outflows to the skeletal muscle and splanchnic organs were increased with resultant decrease in blood flow in these tissues [3, 4]. During anaphylactic hypotension, excitation of the renal nerves was reported in rats [5, 6, 7] and the sympathetic nervous system plays an important protective role [8]; however it is not known whether sympathoexcitation is related to the blood flow change of redistribution during anaphylactic shock. Thus, we hypothesize that anaphylactic hypotension stimulates the redistribution of skeletal muscle blood flow via the excitation of the innervating sympathetic activity. We here examined this issue by focusing on the hindlimb blood flow and the innervating lumbar sympathetic nerve activity (LSNA). The primary aim of this study, therefore, was to determine the changes of the efferent LSNA and femoral arterial blood flow (FBF) during anaphylactic hypotension in anesthetized rats. Secondly, we determined whether LSNA is involved in the FBF response to anaphylaxis. Finally, we revealed the role of the baroreceptor reflex in the responses of LSNA and skeletal hemodynamics to anaphylaxis.

## Material and Methods

### Animals

Male Sprague-Dawley rats, weighing 300-400g, were housed in a room maintained at 24±1°C and illuminated for 12 hours every day. Food and water were freely available. All the animal care and handling procedures were approved by the animal research committee of Kanazawa Medical University.

### Sensitization

Rats in the anaphylaxis, lumbar sympathectomy (LS) and sinoaortic denervation (SAD) groups were sensitized though subcutaneous injection of an emulsion made by mixing equal volumes of complete Freund’s adjuvant (0.5ml) with 1 mg ovalbumin (grade V, Sigma St. Louis, USA) dissolved in physiologic saline (0.5 ml) two weeks before experiment [9].

### Surgery preparation and measurement

Rats were anesthetized with an intraperitoneal injection of pentobarbital sodium (50 mg/kg). The adequacy of anesthesia was monitored by the stability of blood pressure and respiration under control conditions and during a pinch of the hind paw. When a significant change of blood pressure (+5 mmHg) was detected by a paw pinch, additional pentobarbital sodium (5 mg/kg) was intraperitoneally injected. The trachea was intubated to facilitate spontaneous breathing. Polyethylene catheters were inserted into the left femoral vein, right jugular vein and left carotid artery for intravenous injections, central venous pressure (CVP) measurement and systemic arterial pressure (SAP) measurement, respectively. A pulsed Doppler flow probe (MC2PSB; Transonic Systems, Ithaca, NY) was placed on the right femoral artery for continuous measurement of the femoral arterial blood flow (FBF). The animal was placed on a heating pad to maintain the body temperature at 36–37°C. The SAP and CVP were continuously measured with pressure transducers (TP-400T; Nihon-Kohden, Tokyo, Japan), and the reference level was set at the level of the right atrium. Heart rate (HR) was measured by triggering SAP. These hemodynamic variables along with FBF were digitally displayed and recorded by Power Lab (AD Instruments, Castle Hill, Australia). The femoral arterial vascular resistance (FVR) was calculated by the following equation: FVR = (SAP − CVP)/FBF.

LSNA was measured as follows: Following an abdominal mid-line incision at supine position, the intestines were retracted, and the abdominal aorta and vena cava caudal to the renal vessels were pulled aside to expose a lumbar nerve. The left lumbar sympathetic trunk from L3 to L4 supplying skeletal muscle [10, 11] was carefully isolated from peripheral connective tissue using a dissecting microscope and the trunk was not cut but kept intact. The nerve was attached to a pair of homemade stainless steel electrodes. The recording electrodes were fixed with silicon gel to prevent dehydration and electrical noise signals. The electrical change in the nerve was amplified 50,000–100,000 times with a bandpass of 100 to 1000 kHz and monitored by an oscilloscope. Primitive nerve activity data were converted to standard pulse by a window discriminator. Both the discharge rate and the neurogram were sampled with a Power Lab for recording and data analysis on a computer.

**Fig 1:**
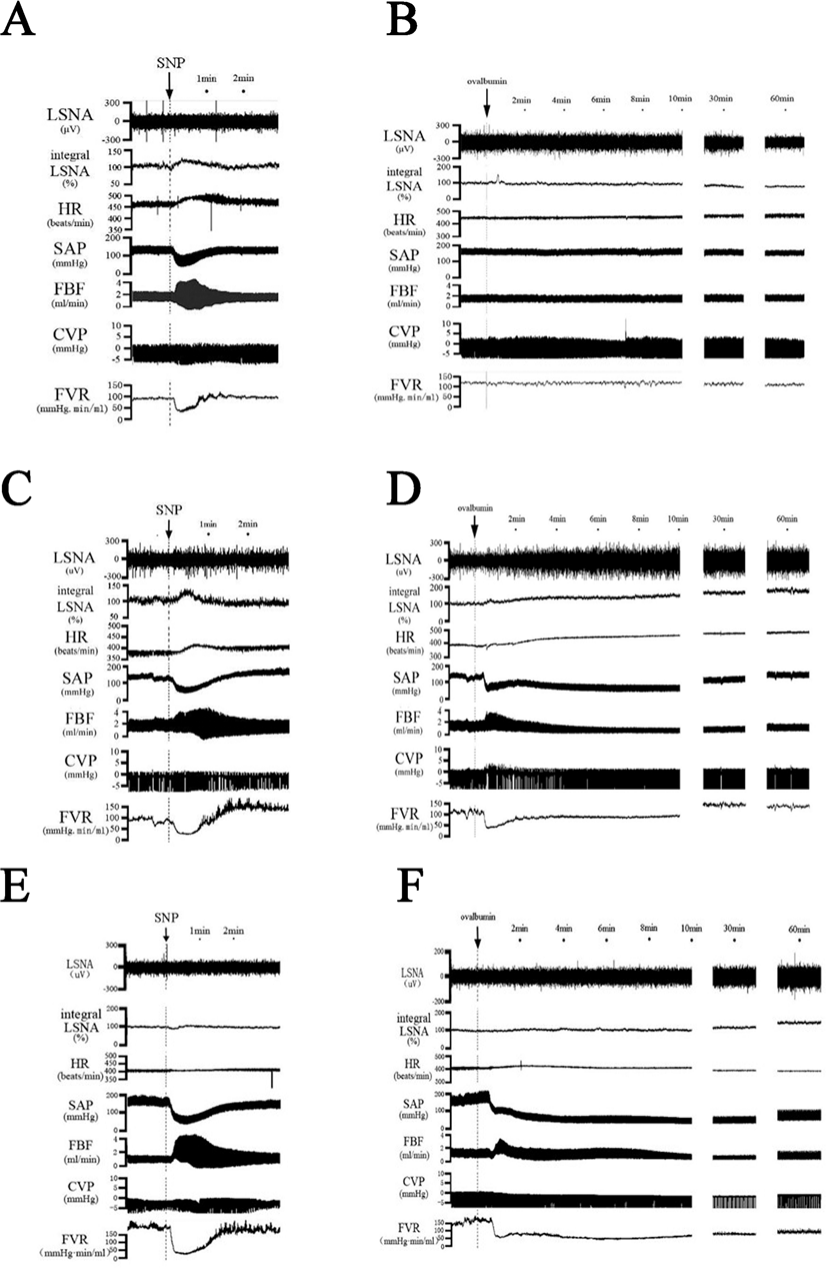
Representative recordings of the sympathetic and hemodynamic responses after an intravenous injection of the SNP or the ovalbumin antigen in a non-sensitized (A; SNP, B; ovalbumin antigen), an anaphylaxis rat (C; SNP, D; ovalbumin antigen) and an anaphylaxis rat with SAD (E; SNP, F; ovalbumin antigen). LSNA, lumbar sympathetic nerve activity; HR, heart rate; SAP, systemic arterial pressure; FBF, femoral arterial blood flow; CVP, central venous pressure; FVR, femoral vascular resistance.

All the rats were allowed to stabilize for 10–30 min after surgery and then sodium nitroprusside (SNP, 30μg/kg) was injected intravenously to confirm baroreceptor-mediated sympathoexcitation. The occurrences of baroreceptor reflex in the non-sensitized group and anaphylaxis group were confirmed by increased LSNA and HR (Fig 1A and 1C). Next, the baseline level was measured for 8–15 min prior to the intravenous injection of the antigen ovalbumin (0.6 mg). The parameters were recorded for 60 min continuously. At the end of the experiment, hexamethonium chloride (10 mg/kg) was intravenously administered to ensure that the post-ganglionic efferent sympathetic nerve activity was recorded. After the experiment, rats were euthanized with an overdose of pentobarbital sodium injected intravenously.

### Bilateral lumbar sympathectomy

In the anaphylaxis-LS group, denervation was accomplished by cutting bilateral lumbar sympathetic nerves from L3 to L4 along the abdominal aorta and vena cava. Immediately the abdominal aorta and vena cava was painted with 10% phenol diluted with ethanol. Careful application of phenol was taken to protect the adjacent tissue from the harmful effects of phenol. The occurrence of baroreceptor reflex in the LS group was confirmed by reflex tachycardia.

### Sino aortic denervation

In the anaphylaxis-SAD group, after the neurovascular trunk in the neck was exposed, the carotid sinus, aortic depressor and recurrent laryngeal nerves were sectioned bilaterally to complete the baroreceptor denervation. Immediately the bilateral carotid arteries were carefully painted with 10% phenol diluted with ethanol to avoid physical and chemical damage to the vagal nerves. The rats were allowed to recover for 30–60 min after denervation surgery. Successful baroreceptor denervation was confirmed by the absence of reflex increases in HR and LSNA in response to the hypotension induced by SNP (Fig 1E).

### Data analysis

All results are expressed as the means ± SE. Comparison of individual values within a group was made by the repeated-measures ANOVA followed by the Fisher post hoc test. Comparison of sympathetic and hemodynamic responses to the antigen between the four groups was made by two way ANOVA with Bonferroni post hoc test. Differences were considered statistically significant at *P* < 0.05.

## Results

### Lumbar sympathetic and hemodynamic responses to anaphylaxis in anesthetized rats

**Fig 2:**
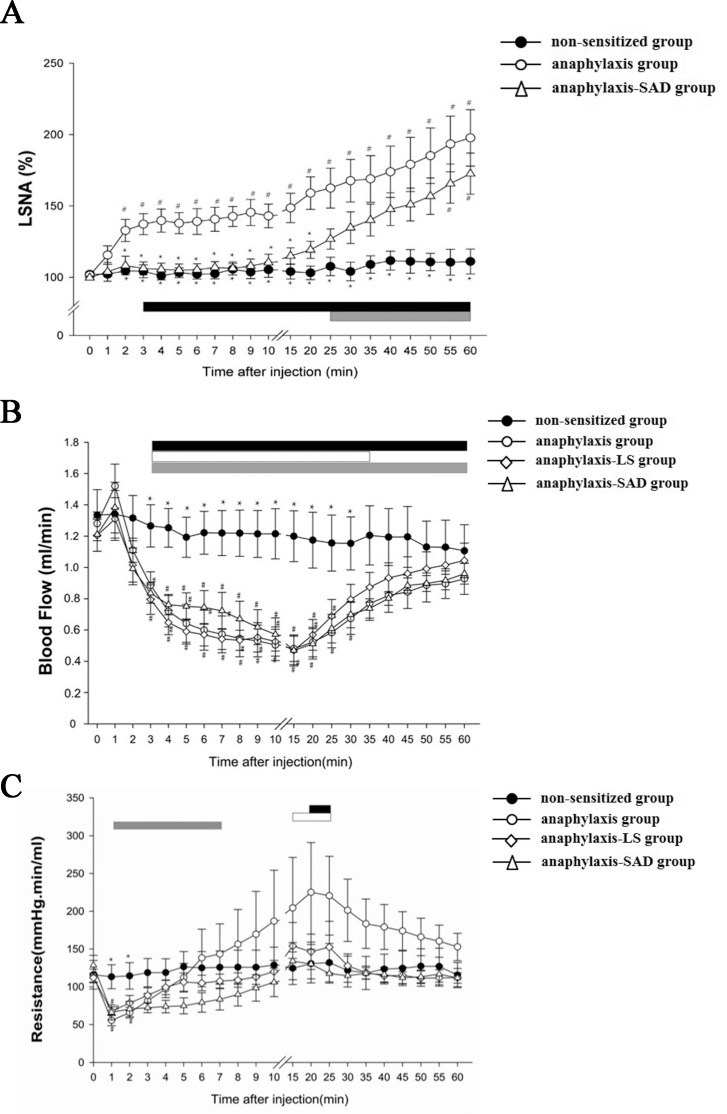
Summary of the LSNA, FBF and FVR changes during anaphylaxis. The time-course data of LSNA (A), FBF (B) and FVR (C) after an intravenous injection of the ovalbumin antigen were shown. ● non-sensitized group; ○ anaphylaxis group; ◇ anaphylaxis-LS group; △ anaphylaxis-SAD group. n = 7 rats per group in this experiment. Values are expressed as means±SE. #P<0.05 vs. the non-sensitized group. *P<0.05 vs. the anaphylaxis group. The black, white and gray bars show the significant differences from the corresponding baseline values in the anaphylaxis group, anaphylaxis-LS group and anaphylaxis-SAD group, respectively.

**Fig 3:**
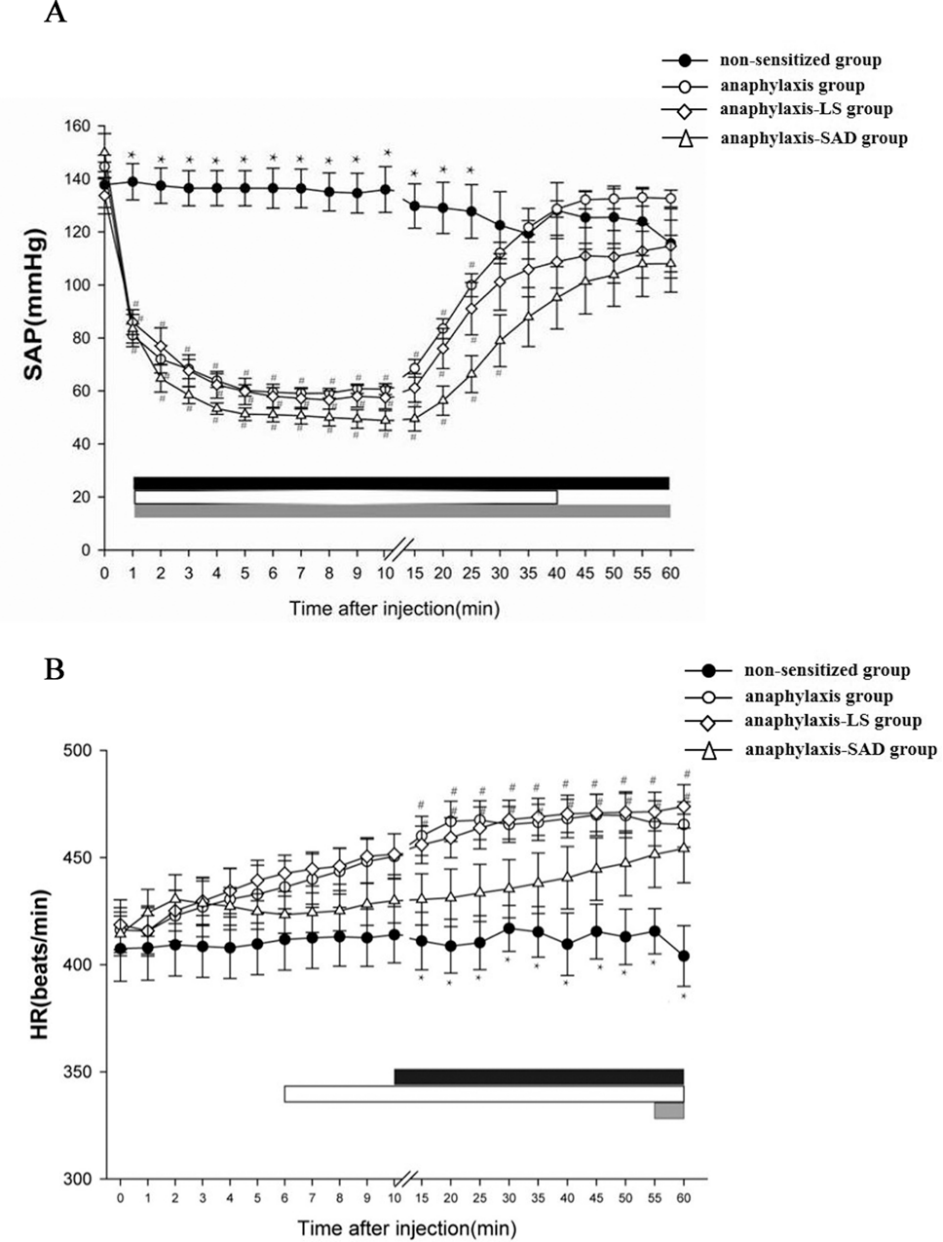
Summary of the hemodynamic changes during anaphylaxis. The time-course data of the SAP (A) and HR (B) after an intravenous injection of the ovalbumin antigen were shown. ● non-sensitized group; ○ anaphylaxis group; ◇ anaphylaxis-LS group; △ anaphylaxis-SAD group. n = 7 rats per group in this experiment. Values are expressed as means±SE. #P<0.05 vs. the non-sensitized group. *P<0.05 vs. the anaphylaxis group. The black, white and gray bars show the significant differences from the corresponding baseline values in the anaphylaxis group, anaphylaxis-LS group and anaphylaxis-SAD group, respectively.

Examples of the response of LSNA, HR, SAP, FBF and CVP to an injection of antigen into a non-sensitized rat and an ovalbumin-sensitized rat were shown in Fig 1 and the summarized data were shown in Figs 2 and 3. In the anaphylaxis group, LSNA was increased significantly to 137.3±7.4% of baseline at 3 min after antigen injection, and remained at this level until 15 min. Thereafter, LSNA progressively increased to 197.7±19.8% of baseline at the end of the experimental period of 60 min (Figs 1D and 2A). In the present study, we recorded LSNA under the intact condition without cutting the nerve. Thus there is a possibility that the responded LSNA included the afferent LSNA. Accordingly, in additional and separate experiments, we recorded afferent LSNA of the sensitized rats with the lumbar sympathetic nerve cut rostrally (n = 5) and found that it did not significantly change when anaphylactic hypotension was evoked: the afferent LSNA measured at 2, 3 and 10 min after antigen injection was 105±4%, 103±4% and 102±5%, respectively, of the baseline (100%). This result indicated that the change in LSNA during anaphylaxis was caused by that of efferent LSNA. FBF transiently increased at 1 min after antigen injection from the baseline of 1.33±0.07 to 1.52±0.14 ml/min in accordance with the start of rapid fall of SAP (Fig 2B). Then FBF was decreased by 63.9% to a nadir of 0.48±0.08 ml/min at 15 min, followed by a gradual recovery to 0.93±0.10 ml/min at 60 min. (Fig 2B). In accordance with the initial increase in FBF, FVR significantly decreased to 50.7% of baseline level from the baseline of 109±7 mmHg・min/mL at 1 min after antigen (Fig 2C). Thereafter FVR recovered and did not change significantly from the baseline level except a slight but significant increase at 20–25 min (Fig 2C). Antigen injection decreased SAP, and increased HR: SAP decreased from the baseline of 144±3 mmHg to a nadir of 59±2 mmHg at 7 min after antigen injection, and then returned gradually to 133±3 mmHg at 60 min (Fig 3A). HR was increased significantly from the baseline of 420±11, reaching 465±10 beats/min at 60 min after antigen injection (Fig 3B). On the other hand, any parameters studied did not change significantly throughout the experimental period in the non-sensitized group (Figs 1B, 2 and 3).

### The effects of sinoaortic denervation on the responses to anaphylaxis in anesthetized rats

In the anaphylaxis-SAD group, LSNA showed no significant changes in response to an antigen injection despite the occurrence of SAP fall during the early phase (0–20 min after antigen injection). There were significant differences in LSNA during this period between the SAD and anaphylaxis groups (Figs 1F and 2A). Thereafter, LSNA in the anaphylaxis-SAD group began to increase progressively in parallel with that of the anaphylaxis group, reaching the peak level of 173±14% of baseline at 60 min (Fig 2A). This finding suggests that LSNA is inhibited by sinoaortic denervation in the early phase of anaphylaxis. On the other hand, the other variables of SAP (Fig 3A), HR (Fig 3B), FBF (Fig 2B) and FVR (Fig 2C) in the anaphylaxis-SAD group responded to anaphylaxis in a manner similar to that in the anaphylaxis group, and no significant differences in these variables were found between the two groups.

### The effects of lumbar sympathectomy

In order to determine the role of LSNA in the responses of FBF (Fig 2B), FVR (Fig 2C) and SAP (Fig 3A) to anaphylaxis, anaphylaxis was evoked in rats with bilateral LS. In response to the antigen, both FBF (Fig 2B) and SAP (Fig 3A) of the anaphylaxis-LS group were decreased similarly to those of the anaphylaxis group, and no significant differences were found between the two groups. FVR of the anaphylaxis-LS group showed changes similar to that of the anaphylaxis group after antigen injection, and there were no significant differences in the time course changes in FVR between the two groups (Fig 2C).

## Discussion

In the present study, we investigated lumbar sympathetic and femoral vascular responses to anaphylactic hypotension in the ovalbumin-sensitized anesthetized rats, and obtained the following findings: 1) After the antigen injection, LSNA was increased and FBF was substantially decreased, while FVR initially decreased and then returned to the baseline. 2) SAD inhibited the antigen-induced activation of LSNA only at the early phase. 3) Neither SAD nor LS affected the responses of FVR, FBF or SAP to anaphylaxis. These findings indicate that LSNA does not participate in regulation of the blood flow to hindquarter skeletal muscles during anaphylactic hypotension in anesthetized rats. To the best our knowledge, this is the first experimental study to clarify, by measuring simultaneously the femoral arterial blood flow and LSNA, the relationship between the blood flow to hindlimb or hindquarter skeletal muscles and their relevant sympathetic outflow during anaphylactic hypotension.

### The changes of LSNA during anaphylactic shock

Previously, it was reported that rat anaphylactic shock activated the sympathetic nervous system, as evidenced by the elevated plasma concentrations of epinephrine and norepinephrine [8, 12] and increased renal sympathetic nerve activity [5, 6]. In the present study, we further demonstrated that LSNA was also increased during anaphylaxis in the anesthetized rats. The increase in LSNA in the early phase of anaphylactic hypotension may be ascribed to the baroreceptor reflex, because SAD abolished the anaphylaxis-induced sympathoexcitation. However, the mechanism of the increase in LSNA in the late phase is not known. We assume that activation of brain stem chemoreceptors by acidosis during anaphylaxis-induced sustained hypotension might account for the sympathoexcitation [13, 14]. Another possibility is that chemical mediators produced during anaphylaxis [15], such as histamine and prostaglandins directly activate the sympathetic nervous system.

In contrast to the dependency of the sympathoexcitation of LSNA via the baroreceptor reflex, the anaphylaxis-induced increase in renal sympathetic nerve activity was not affected by SAD in our previous study [6]. In addition, the hepatic sympathetic nerve activity did not change significantly after the onset of anaphylactic hypotension in the anesthetized rats [8]. Thus, the regionally different responses of sympathetic nerve outflows to the kidney, the hindlimb and the liver during anaphylaxis are triggered and regulated by distinct pathways. Further study is required to determine the mechanisms for the regional difference of sympathetic responses to anaphylaxis.

### Role of LSNA in regulation of the femoral vascular tone during anaphylactic shock

Generally, during severe hypotension, as observed in all forms of systemic shock, the blood flow to peripheral organs such as skeletal muscle is reduced to keep constant blood flow supplied to vital organs such as brain and heart. In fact, skeletal muscle blood flow in anesthetized rats was decreased under the anaphylactic shock [16]. To redistribute the systemic blood flow, the sympathetic nerve activity was increased so as to induce vasoconstriction of the innervating vascular beds, resulting in reduction of blood flow. Actually, previous studies demonstrated that lumbar sympathetic nerves innervating the hindlimb play an important role in regulating skeletal vasomotion [17, 18]. Consistent with this notion, in the present study, anaphylaxis caused both a decrease in FBF supplied to the skeletal muscles of the hindlimb and an increase in LSNA. Unexpectedly, however, the selective sympathectomy of the lumbar nerves did not affect the anaphylaxis-induced reduction of FBF. These findings indicate that the increased LSNA does not substantially contribute to the reduction of FBF during the anaphylactic hypotension in the present anesthetized rats. We may explain this unexpected result as follows: 1) The vasoconstrictors released during anaphylaxis overwhelm the vasoconstrictive effects of increased LSNA. Our previous findings that the plasma concentrations of vasoconstrictive hormones such as norepinephrine, vasopressin and angiotensin II were substantially increased after antigen injection strongly support this possibility [7, 19]. Obviously, systemic vasoconstrictive mediators such as thromboxane A_2_ [20] and leukotriene C_4_ [21] were also released systemically from mast cells and basophils [15, 22]. 2) The responsiveness of the femoral arteries might be reduced to norepinephrine released from the sympathetic nerve endings during anaphylaxis in the present study. It is reported that nitric oxide derived by neuronal nitric oxide synthase and endothelial nitric oxide synthase could inhibit sympathetic vasoconstriction in resting skeletal muscles of the anesthetized rats [23]. Actually, nitric oxide is produced during anaphylaxis [24, 25]. 3) The blood flow to the hindlimb was already maximally reduced due to the anaphylaxis-induced reduction of the venous return or cardiac output [7]. Therefore there might have been no room for the lumbar sympathoexcitation to further reduce FBF through induced vasoconstriction.

### The regulation of systemic arterial pressure and blood flow during anaphylactic shock

An interesting finding of the present study is that the femoral arterial vasodilatation, as evidenced by increased FBF and decreased FVR, occurred immediately after the antigen injection, and simultaneously with the initial fall of SAP. The similar vasodilatation is observed in the arteries of other vascular beds, such as the hepatic artery and mesenteric artery during anaphylaxis in anaesthetized rats [26, 27]. This vasodilatation was quickly followed by return to the basal levels [26, 27], as observed in the present study. We assume that the initially occurring vasodilatation just triggers the rapid fall of SAP.

The blood flow to the hindlimb was decreased by 63.9% in the present study. This reduction of FBF was much smaller than that of the splanchnic vascular beds as observed in our previous studies, in which the same rat anaphylaxis models were used: the blood flow reductions of the mesenteric artery [26] and the portal veins [8] were 84% (from 11.8±0.8 to 1.9±0.3 ml/min) and 82% (from 27.0±2.3 to 4.8±0.8 ml/min), respectively. The other investigators also reported that the blood flow to the small bowel decreased more than 90% in rat anaphylactic shock [28]. These lines of evidence suggest that the role of skeletal muscles of the hindlimb in mobilization of blood is smaller than that of the splanchnic vascular beds during anaphylactic shock.

In summary, the present study shows that anaphylactic hypotension in anesthetized rats is accompanied by FBF reduction and lumbar sympathoexcitation which depends on the baroreceptor reflex only in the early phase of anaphylaxis. However, the FBF and SAP responses were not mediated by either the lumbar sympathetic nerves or the baroreceptor reflex. Thus, we conclude that lumbar sympathetic nerves are not critical to regulation of the blood flow to the hindlimb or blood pressure during anaphylactic hypotension in anesthetized rats.

## Abbreviations

LSNA: lumbar sympathetic nerve activity
FBF: femoral blood flow
LS: lumbar sympathectomy
SAD: sinoaortic denervation
SAP: systemic arterial pressure
HR: heart rate
CVP: central venous pressure
FVR: femoral arterial vascular resistance
SNP: sodium nitroprusside
